# A Coxsackievirus B vaccine protects against virus-induced diabetes in an experimental mouse model of type 1 diabetes

**DOI:** 10.1007/s00125-017-4492-z

**Published:** 2017-11-18

**Authors:** Virginia M. Stone, Minna M. Hankaniemi, Emma Svedin, Amirbabak Sioofy-Khojine, Sami Oikarinen, Heikki Hyöty, Olli H. Laitinen, Vesa P. Hytönen, Malin Flodström-Tullberg

**Affiliations:** 1 The Center for Infectious Medicine, Department of Medicine, Karolinska Institutet, Karolinska University Hospital Huddinge, F59, SE-141 86 Stockholm, Sweden; 2 Faculty of Medicine and Life Sciences, University of Tampere, Tampere, Finland; 3 Fimlab Laboratories, Pirkanmaa Hospital District, Tampere, Finland

**Keywords:** Antibody, Coxsackievirus, Enterovirus, Mouse model, NOD mice, Type 1 diabetes, Vaccine

## Abstract

**Aims/hypothesis:**

Epidemiological studies suggest a role for Coxsackievirus B (CVB) serotypes in the pathogenesis of type 1 diabetes, but their actual contribution remains elusive. In the present study, we have produced a CVB1 vaccine to test whether vaccination against CVBs can prevent virus-induced diabetes in an experimental model.

**Methods:**

NOD and *SOCS1-*tg mice were vaccinated three times with either a formalin-fixed non-adjuvanted CVB1 vaccine or a buffer control. Serum was collected for measurement of neutralising antibodies using a virus neutralisation assay. Vaccinated and buffer-treated mice were infected with CVB1. Viraemia and viral replication in the pancreas were measured using standard plaque assay and PCR. The development of diabetes was monitored by blood glucose measurements. Histological analysis and immunostaining for viral capsid protein 1 (VP1), insulin and glucagon in formalin-fixed paraffin embedded pancreas was performed.

**Results:**

The CVB1 vaccine induced strong neutralising antibody responses and protected against viraemia and the dissemination of virus to the pancreas in both NOD mice (*n* = 8) and *SOCS1-*tg mice (*n* = 7). Conversely, 100% of the buffer-treated NOD and *SOCS1-*tg mice were viraemic on day 3 post infection. Furthermore, half (3/6) of the buffer-treated *SOCS1-*tg mice developed diabetes upon infection with CVB1, with a loss of the insulin-positive beta cells and damage to the exocrine pancreas. In contrast, all (7/7) vaccinated *SOCS1-*tg mice were protected from virus-induced diabetes and showed no signs of beta cell loss or pancreas destruction (*p* < 0.05).

**Conclusions/Interpretation:**

CVB1 vaccine can efficiently protect against both CVB1 infection and CVB1-induced diabetes. This preclinical proof of concept study provides a base for further studies aimed at developing a vaccine for use in elucidating the role of enteroviruses in human type 1 diabetes.

**Electronic supplementary material:**

The online version of this article (10.1007/s00125-017-4492-z) contains peer-reviewed but unedited supplementary material, which is available to authorised users.

## Introduction

Type 1 diabetes is increasing globally, however, the mechanisms that initiate the onset of disease remain unknown. Environmental factors that have been implicated include enterovirus infections, in particular Coxsackievirus B (CVB) serotypes [1, 2]. CVBs are common RNA viruses encompassing six serotypes; CVB1–6. Usually infections are mild or asymptomatic, however, some lead to severe, potentially fatal diseases including aseptic meningitis and myocarditis. Epidemiological studies exist documenting associations between CVBs and type 1 diabetes, including the presence of virus in the pancreas of individuals recently diagnosed with type 1 diabetes and increased occurrence of infection prior to islet antibody appearance and onset of diabetes [1–3]. Furthermore, CVBs can induce hyperglycaemia in animal models [4, 5]. Together, these findings support the hypothesis that CVBs contribute to the pathogenesis of type 1 diabetes.

Despite the aforementioned circumstantial evidence, it is possible that the association between CVBs and type 1 diabetes is not causal and results from yet unidentified confounding factors. Vaccine studies in prospective birth cohorts of genetically susceptible children may help elucidate the role of CVBs in type 1 diabetes, however, no commercially available CVB vaccines currently exist [2]. We previously demonstrated that an inactivated, non-adjuvanted CVB1 vaccine was well tolerated in mice, was highly efficacious against CVB1 infection and did not accelerate diabetes in NOD mice [6, 7]. Whether this vaccine can prevent CVB1-induced diabetes is unknown.

*SOCS1*-tg mice express the suppressor of cytokine signalling-1 in beta cells, resulting in their inability to respond to interferons, thus leaving beta cells susceptible to CVB infection, destruction and subsequently virus-induced diabetes [4]. Due to its robustness and quick development of virus-induced diabetes, the *SOCS1-*tg mouse model was used to assess the ability of a CVB1 vaccine to prevent CVB1 induced diabetes, providing an important biological proof of concept study examining CVB vaccine efficacy in the context of type 1 diabetes.

## Methods

### Animal husbandry and monitoring of animal health

NOD mice and *SOCS1-*tg mice on a NOD background (both from in-house breeding) were housed in specific pathogen-free conditions at Karolinska Institutet, Stockholm, Sweden. Ethics approval was granted for all experiments by the local ethics committee and were conducted in accordance with the NIH Principles of Laboratory Animal Care and national laws in Sweden. Extended health monitoring of mice was performed. Additional information is provided in the ESM Methods: Animals, together with details of primers used for *SOCS1-*tg mouse genotyping (ESM Table 1).

### Virus and vaccine production

A CVB1 field isolate (CVB1-V200; [3]), was propagated in Vero cells (ECACC no. 84113001; mycoplasma negative), purified and then formalin inactivated for 3 days at 37°C to produce CVB1 vaccine. See ESM Methods: Vaccine production and Hankaniemi et al [7] for more details. Mice were infected with CVB1-10796 (propagated in HeLa cells, originally obtained from R Glas, Karolinska Institutet, Stockholm, Sweden, mycoplasma negative).

### Vaccinations

**Fig. 1:**
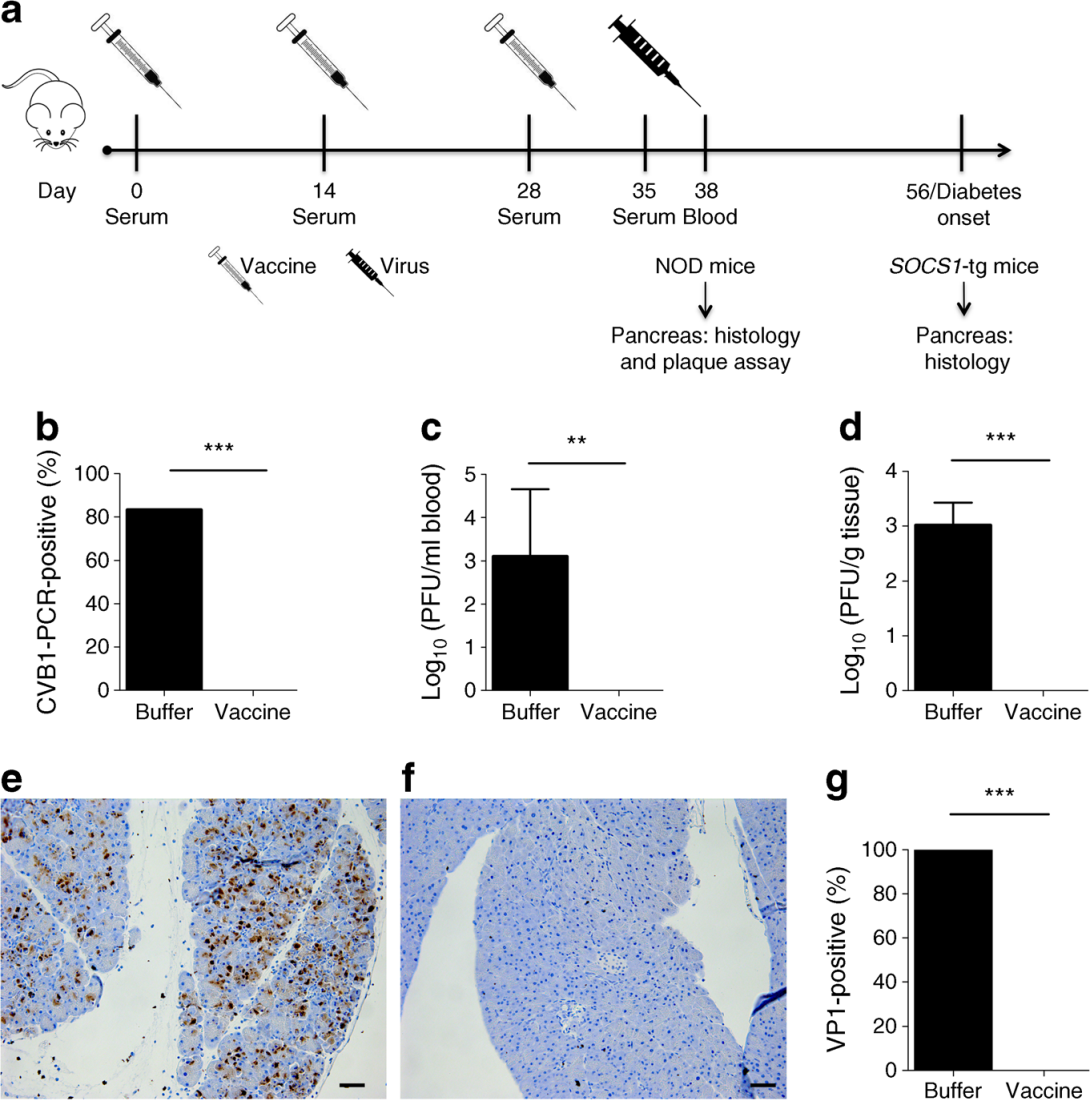
A CVB1 vaccine protects NOD mice against viraemia and systemic viral spread following infection with CVB1. (**a**) Schematic illustrating the experimental timeline; NOD mice and *SOCS1*-tg mice were vaccinated or given buffer alone, followed by infection with CVB1. For NOD mice, the experiments were terminated on day 3 p.i. Mock vaccinations and CVB1 vaccinations are represented by the empty syringe; CVB1 challenge is shown with the black syringe. (**b**) Percentage of infected vaccinated (*n* = 8) or buffer-treated (*n* = 6) mice determined by the presence of CVB1 RNA in the blood of NOD mice on day 3 p.i. as detected by RT-PCR. ***p < 0.001, *χ* ^2^ test. (**c**) Cytopathic virus in the blood and (**d**) in the pancreas on day 3 p.i. in NOD mice treated with buffer (*n* = 6) or CVB1 vaccine (*n* = 8) as measured by standard plaque assay. Mean values ± SD; **p < 0.01 and ***p < 0.001, Mann–Whitney *U* test. (**e**) Representative images of VP1 positivity (brown staining) in pancreas sections of buffer-treated and (**f**) CVB1-vaccinated NOD mice on day 3 p.i. (×16 magnification; scale bar, 50 μm) and (**g**) percentage of mice with VP1 positivity in the pancreas. ***p < 0.001, *χ* ^2^ test

Male and female age-matched NOD and *SOCS1-*tg mice (4–7 weeks old) were randomly assigned to groups and vaccinated on days 0, 14 and 28 with non-adjuvanted vaccine containing 1.8 μg protein (*n* = 8 and *n* = 7, respectively) or mock-vaccinated with vaccine buffer (M199-0.1% Tween80 (vol./vol.), 150 μl, *n* = 6 for NOD and for *SOCS1-*tg mice) by interscapular injection [6, 7]. Serum was collected before vaccinations and infection (day 35) by tail bleeding (experimental timeline displayed in Fig. 1a). The study was not blinded to the experimenter.

### Infections

Mice were challenged with 10^6^ plaque forming units (PFU) CVB1-10796 (diluted in serum-free RPMI to a final volume of 200 μl, administered by intraperitoneal injection, with the dose carefully optimised, data not shown) on day 35. Blood samples were collected on day 3 post infection (p.i.; 1:1 ratio with 12 mmol EDTA). NOD mice were killed on day 3 p.i. and pancreases saved for virus quantification and histological analysis. *SOCS1-*tg mice were monitored until diabetes development or day 21 p.i. and pancreases saved for histological analysis.

### Monitoring of blood glucose and diabetes development

Blood glucose was measured in blood obtained from the tail-vein with a Bayer Contour XT blood glucose meter (Bayer, Basel, Switzerland) and a glucose concentration >18 mmol/l, or two consecutive measurements between 13 and 18 mmol/l were used to define diabetes and diabetic mice were killed.

### Neutralising antibody detection

Neutralising antibody titres were determined by standard virus plaque reduction assay in green monkey kidney (GMK) cells (National Institute for Health and Welfare, Finland; mycoplasma negative) [3]. Plaque number reduction ≥80% compared with untreated virus suspension was considered positive. The detection limit of the assay was a fourfold dilution (1:4), and positivity cut-off for serum samples was set to ≥1:16.

### Virus titration

Pancreases were homogenised and lytic virus quantified in either blood or pancreas by standard plaque assay in GMK cells. Viral titres are expressed as PFU/g of tissue or ml of blood. See ESM Methods: Virus titration and tissue homogenisation for more details.

### PCR analysis

Enterovirus specific real-time PCR was performed using RNA extracted from blood samples; see ESM Methods: PCR analysis and Honkanen et al [8] for further details. Primers are shown in ESM Table 2.

### Histological analysis and immunohistochemistry

Histological analysis and immunohistochemistry of pancreas samples with viral capsid protein 1 (VP1), insulin and glucagon were carried out as in Flodström et al and Larsson et al ([4, 6]; ESM Methods: Immunohistochemistry and antibodies).

### Statistical analysis

Statistical analyses were executed using Prism 5 software (GraphPad, La Jolla, CA, USA). PCR and VP1 immunohistochemistry data were analysed by *χ* ^2^ tests. Plaque assay virus titrations were analysed by Mann–Whitney *U* test. Neutralising antibody data was analysed by one-way ANOVA. Diabetes incidence was analysed by log rank Mantel–Cox test. Data are expressed as the mean ± SD. A *p* value ≤0.05 was regarded statistically significant.

## Results

### CVB1 vaccine is well tolerated and is highly immunogenic

The newly produced CVB1 vaccine was well tolerated by NOD mice with no adverse effects on weight or blood glucose (ESM Fig. 1a–c). Furthermore, the vaccine was highly immunogenic and vaccinated mice produced CVB1 neutralising antibodies after the primary immunisation, which was augmented after the second immunisation (ESM Fig. 1d). Serum with a neutralising capacity was not detected in buffer-treated mice (data not shown).

### CVB1 vaccine protects against CVB1 infection in NOD mice

We next examined whether the vaccine protects against viraemia caused by CVB1 infection and prevents virus replication in the pancreas on day 3 p.i. All vaccinated mice (8/8) were protected from viraemia, as determined by RT-PCR and plaque assay (Fig. 1b, c). Conversely, all buffer-treated mice were identified viraemic by plaque assay (Fig. 1c) and 5/6 were positive for CVB1 RNA (Fig. 1b). Similarly, replicating virus in the pancreas was measured in buffer-treated mice but not in vaccinated mice (Fig. 1d). Immunohistochemical analysis using the VP1 antibody further confirmed viral dissemination to the pancreas in all buffer-treated mice (Fig. 1e, g) but not vaccinated mice (Fig. 1f–g).

### CVB1 vaccine protects against virus-induced diabetes

**Fig. 2:**
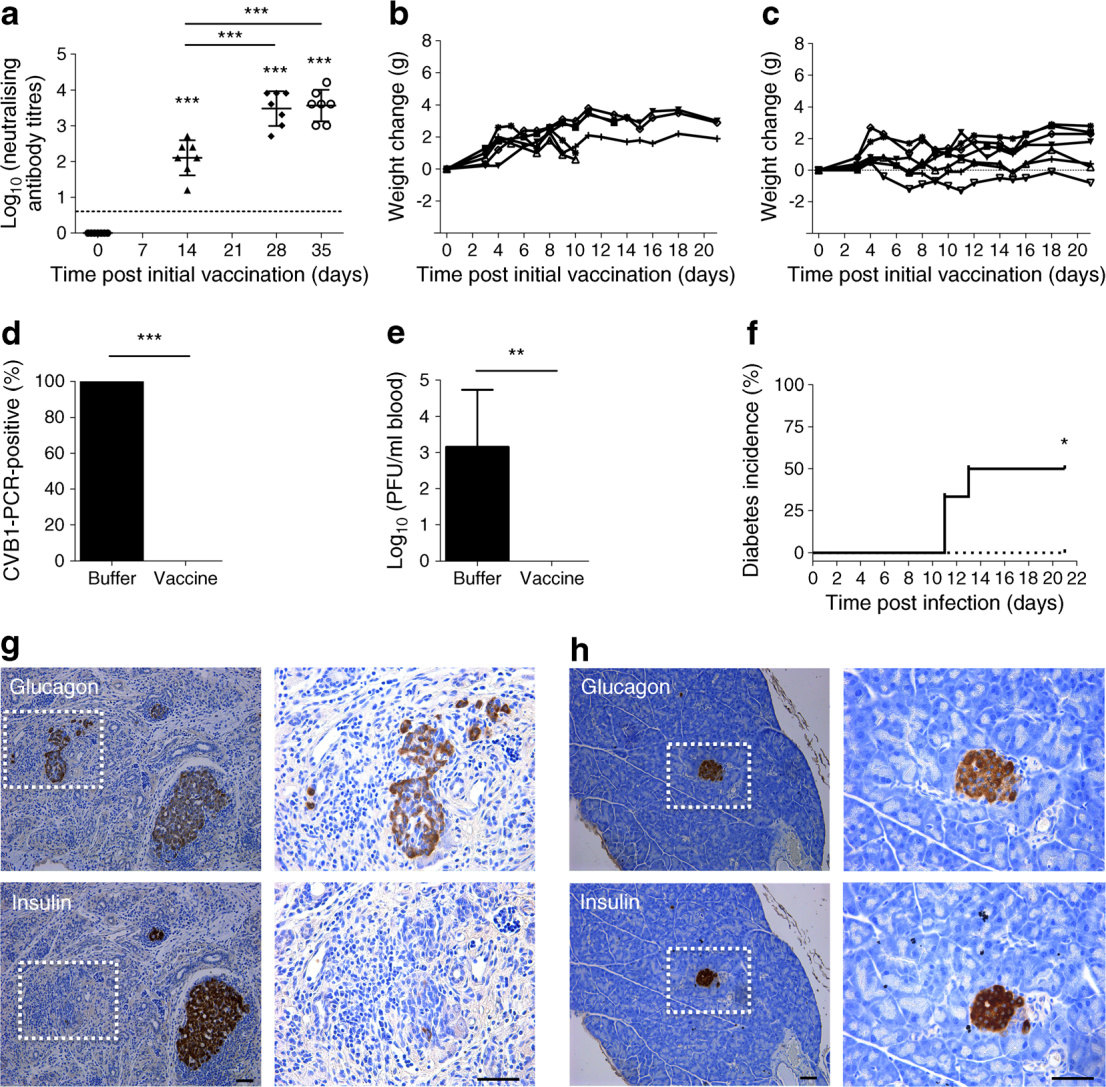
*SOCS1-*tg mice are protected from virus-induced diabetes by the CVB1 vaccine. (**a**) Neutralising antibody titres in the serum of vaccinated mice (*n* = 7) sampled prior to vaccination on days 0, 14 and 28 and before infection on day 35. The dotted line illustrates the neutralising capacity threshold in the virus neutralisation assay. Each serum sample was analysed in two independent neutralisation assays and the mean neutralising antibody titre calculated. Mean values are indicated by the line ± SD; ***p < 0.001 compared with day 0 or indicated time point as determined by one-way ANOVA. (**b**, **c**) Weight changes of individual mice treated with vaccine buffer (*n* = 6) (**b**), or CVB1 vaccine (*n* = 7) (**c**) after infection with 10^6^ PFU CVB1. Each individual animal is represented by a single line. Three of the buffer-treated animals developed diabetes and were removed prior to day 21. (**d**) Percentage of buffer-treated (*n* = 6) or vaccinated mice (*n* = 7) positive for CVB1 in the blood on day 3 p.i. as detected by RT-PCR. ***p < 0.001, *χ* ^2^ test. (**e**) Cytopathic virus measured in the blood of buffer-treated (*n* = 6) or vaccinated (*n* = 7) mice on day 3 p.i. by standard plaque assay. Mean values ± SD; **p < 0.01, Mann–Whitney *U* test. (**f**) Cumulative diabetes incidence in buffer-treated (black line) and vaccinated (dotted line) *SOCS1-*tg mice after infection with CVB1, *p* < 0.05 comparing the two groups as determined by logrank Mantel–Cox test. Formalin-fixed, paraffin embedded *SOCS1-*tg mice pancreas sections stained with insulin or glucagon antibodies by immunohistochemistry. Shown are representative images from (**g**) buffer-treated and (**h**) CVB1-vaccinated mice. Images on the left of each panel are at ×16 magnification and the white box indicates the area of magnification shown in the right panels (at ×40 magnification). Scale bars, 50 μm. (**g**) Note the loss of acinar tissue and immune cell infiltration in tissue from buffer-treated animals

*SOCS1-*tg mice, which are susceptible to virus-induced diabetes [4], showed no adverse changes in weight and blood glucose after CVB1 vaccination (data not shown). Additionally, vaccinated *SOCS1-*tg mice developed a robust antibody response (Fig. 2a) similar to that observed in NOD mice (ESM Fig. 1d). Buffer-treated mice remained consistently negative for neutralising antibodies (data not shown).

We next monitored *SOCS1-*tg mice after CVB1 challenge. No obvious differences were found in the weight of vaccinated and buffer-treated mice (Fig. 2b, c). Furthermore, viraemia measurements on day 3 p.i. revealed no signs of infection in the vaccinated animals (0/7; Fig. 2d, e). In contrast, all (6/6) buffer-treated mice were infected as indicated by the detection of both viral RNA (Fig. 2d) and infective virus by plaque assay (Fig. 2e).

We also tracked diabetes development in the infected *SOCS1-*tg mice until day 21 p.i. As expected, diabetes occurred in the buffer-treated *SOCS1-*tg mice with 50% (3/6) developing hyperglycaemia (*p* < 0.05; Fig. 2f). Pancreatic exocrine damage was notable in 4/6 mice (Fig. 2g), which corresponded with diabetes development. Moreover, mice that developed hyperglycaemia showed glucagon positivity but a loss of insulin positivity in a number of islets, indicating destruction of the insulin-producing beta cells (Fig. 2g). In contrast, all seven vaccinated *SOCS1-*tg mice were protected from diabetes (Fig. 2h) and showed normal pancreas morphology on day 21 p.i. with healthy exocrine tissue and intense insulin and glucagon staining in the islets of Langerhans (Fig. 2h).

## Discussion

In the present study, we show that a monovalent, formalin-inactivated and non-adjuvanted CVB1 vaccine protects against both acute CVB1 infection and virus-induced diabetes in a mouse model for virus-induced diabetes. The vaccine proved to be highly immunogenic, with the antibody titres produced being greater than those considered to be protective in other enterovirus vaccines [9] and was well tolerated with regards to weight and blood glucose. Combined, these results highlight the potential of enterovirus vaccines in testing the hypothesis that preventing enterovirus infections attenuates the risk of type 1 diabetes.

When considering enterovirus vaccine development for clinical intervention trials, it is pertinent to identify enteroviruses with possible roles in type 1 diabetes pathogenesis. Large-scale prospective studies including the Type 1 Diabetes Prediction And Prevention Project (DIPP) and The Environmental Determinants of Diabetes in the Young (TEDDY) Study [2, 10] are therefore highly important owing to their potential in the identification of diabetogenic viruses from clinical samples collected. Moreover, if an enterovirus vaccine were successfully approved for clinical use, prospective studies like these would provide excellent opportunities to test vaccine efficacy in the prevention of type 1 diabetes. Theoretically, traditional formalin-inactivated vaccines could include several different enterovirus types. For example, polio vaccine contains poliovirus 1-3 and recently, a 50-valent rhinovirus vaccine (both enteroviruses) had high immunogenicity in nonhuman primates [11]. However, the economic feasibility of commercial vaccine production currently limits the number of serotypes that can be included in a single vaccine. Existing information suggests the importance of CVB enteroviruses as targets for vaccines to use in type 1 diabetes prevention trials and future studies should aim to produce a hexavalent vaccine including all six CVB serotypes [2]. Moreover, this type of vaccine would be valuable in the prevention of other potentially fatal CVB associated diseases, including myocarditis and aseptic meningitis.

In conclusion, this proof of concept study demonstrates that a formalin-inactivated CVB vaccine protects against virus-induced diabetes. This provides a model for future development of enterovirus vaccines, particularly multivalent vaccines covering a number of serotypes for testing in clinical trials to examine their ability to prevent human type 1 diabetes.

## Electronic supplementary material

ESM (PDF 170 kb)

## Abbreviations

CVB: Coxsackievirus B
GMK: Green monkey kidney
PFU: Plaque forming units
p.i.: Post infection
VP1: Viral capsid protein 1
