# Supplementary material for: A Coxsackievirus B vaccine protects against virus-induced diabetes in an experimental mouse model of type 1 diabetes
Source: Diabetologia. 2017 Nov 18;61(2):476–81. doi: 10.1007/s00125-017-4492-z (PMC6448957; doi:10.1007/s00125-017-4492-z)
Supplement: Supplementary file 1 — (PDF 170 kb) [file 125_2017_4492_MOESM1_ESM.pdf]

Electronic Supplemental Material (ESM) for

A Coxsackievirus B vaccine protects against virus-induced diabetes in an experimental mouse model of type 1 diabetes

Authors: Virginia M. Stone, Minna M. Hankaniemi, Emma Svedin, Amirbabak Sioofy-Khojine, Sami Oikarinen, Heikki Hyöty, Olli H. Laitinen, Vesa P. Hytönen, Malin Flodström-Tullberg

Contents:

Contents: 1

ESM Methods 2

Animals 2

Vaccine production 2

Virus titration and tissue homogenisation 3

PCR analysis 3

Immunohistochemistry and antibodies 3

ESM Table 1 – Primers used for *SOCS1*-tg mouse genotyping. 4

ESM Table 2 – Primers used for the enterovirus-specific real-time PCR 4

ESM Fig. 1: A formalin-fixed CVB1 vaccine is well-tolerated by NOD mice and is highly immunogenic. 5

ESM References: 6

ESM Methods

Animals

Animals were housed in ventilated cages in an SPF unit at the Karolinska University Hospital Huddinge, Stockholm, and provided with water and food ad libitum. Animals were not single housed and a maximum of 5 mice were housed in the same cage.

SOCS1-tg NOD mouse generation, breeding and screening is described by Flodström *et al* (2002) in [1]. Briefly, *SOCS1*-tg mice were bred by crossing heterozygous *SOCS1*-tg mice with NOD mice. The offspring were genotyped to identify the transgenic (*SOCS1*-tg) and non-transgenic (NOD) mice (primers given in ESM Table 1 and named as *hbs* primer 1 and 2 as they amplify part of the *hbs* transgene, inserted into the human insulin promoter/*SOCS1*-construct used to generate the *SOCS1*-tg animals).

Mice were anaesthetised with isofluorane, prior to heart puncture and blood drawing, and then killed by cervical dislocation.

Vaccine production

Vaccine was produced as described in [2]. Briefly, CVB1-V200 was grown in Vero cells, purified by 30% (wt/vol.) and 30/50% (wt/vol.) sucrose pelleting in the presence of 0.1% Tween80 (vol./vol.) and inactivated in 0.01% (vol./vol.) formalin for 3 days at 37°C. Inactivation levels were confirmed by plaque assay titrations. Prior to injection, inactivated CVB1 was diluted in M199-0.1% Tween80 (vol./vol.; vaccine buffer).

Virus titration and tissue homogenisation

Tissues were homogenized using sterile ceramic beads (2.8mm, QIAGEN, Hilden, Germany) by vigorous shaking with a PowerLyzer 24 Bench Top Homogenizer (MoBio Laboratories, QIAGEN) device for three 45 second repeats at 3200rpm. Lytic virus was measured in samples by standard plaque assay in GMK cells and viral titres expressed as PFU/g of wet tissue or ml of blood. Samples were analysed on two separate occasions and the mean values used.

PCR analysis

Blood samples were diluted 1:60 in Hanks solution. RNA was extracted from 140µl diluted blood sample with QIAmp RNA Blood Mini Kit (QIAGEN, Germany). Enterovirus specific real-time PCR and product amplification was performed as described in [3] with QuantiTect Probe kit (QIAGEN, Germany) and Taqman chemistry according to the manufacturer's protocol. The primers (which amplify the 5' non coding region of all known enterovirus serotypes) and probes used are shown in ESM Table 2. The PCR reaction was run in an ABI 7900HT machine (Fisher Scientific, Vantaa, Finland). All samples were run in triplicate. CVB3 and water were used as positive and negative controls respectively.

Immunohistochemistry and antibodies

VP1 was detected by immunohistochemistry using a biotinylated anti-VP1 antibody (5D8/1; DAKO, Ely, UK; biotinylated by Capra Bioscience, Ängelholm, Sweden, 0.07µg/ml final concentration, Tris EDTA pH6 antigen retrieval). The anti-VP1 antibody was biotinylated as it is raised in mouse, and biotinylation circumvents the need for a secondary antibody (which in this case would bind to mouse

immunoglobulins in mouse tissue, leading to unspecific staining). The biotinylated anti-VP1 antibody was validated in infected mouse pancreas known to be VP1 positive. Insulin (1:10,000, A0564, Dako, Ely, UK) and glucagon (1:3000, A0565, Dako, Glostrup, Denmark) staining were carried out as in [1] and both antibodies were validated in mouse pancreas sections. Secondary antibody alone acted as a negative control for insulin (goat-anti guinea pig, Vector Laboratories, Burlingame, CA, USA) and glucagon (goat-anti rabbit, Dako, Glostrup, Denmark) and 2% NGS acted as the negative control for the VP1 experiments.

ESM Table 1 – Primers used for *SOCS1*-tg mouse genotyping.

**Primer**	**Sequence**
hbs 1	GGT CTC CCC GTC TGT GCC TTC TCA
hbs 2	TTG CAT GGT GCT GGT GCG CAG ACC

ESM Table 2 – Primers used for the enterovirus-specific real-time PCR as described by Honkanen *et al* 2013 [3].

**Primer**	**Sequence**
Forward	CGG CCC CTG AAT GCG GCT AA
Reverse	GAA ACA CGG ACA CCC AAA GTA
Probe 1	FAM-TCT GTG GCG GAA CCG ACT A-TAMRA
Probe 2	FAM-TCT GCA GCG GAA CCG ACT A-TAMRA

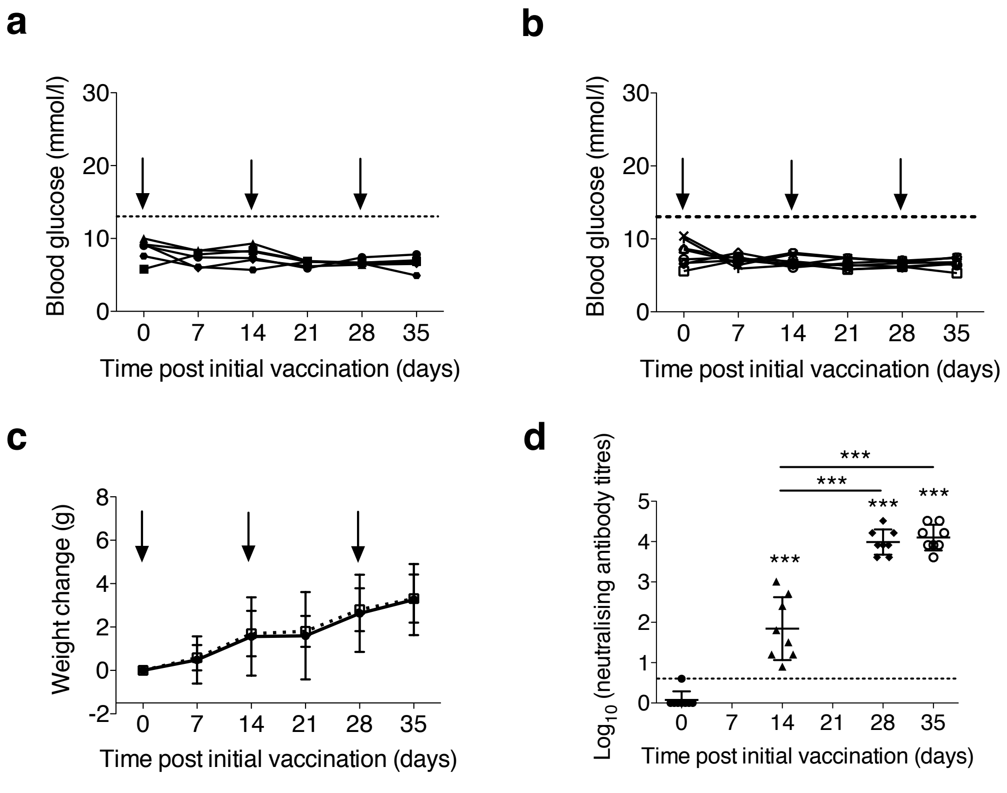

ESM Fig. 1: A formalin-fixed CVB1 vaccine is well-tolerated by NOD mice and is highly immunogenic.

Male and female NOD mice (5-7 weeks of age) were vaccinated or given buffer alone, according to the schedule outlined in Fig. 1a. (a-c) Individual mouse blood glucose levels (a, b) and average weight changes (c) in NOD mice after treatment with vaccine buffer alone (a, c; n=6, solid line) or CVB-1 vaccine on days 0, 14 and 28 (b, c; n=8; dashed line in c). Weight changes (c) are plotted as the mean ± S.D. Black arrows indicate vaccination time points and the dotted lines in (a) and (b) show the diabetes threshold. (d) Serum was collected from vaccinated mice (n=8) prior to vaccination on days 0, 14, 28 and before infection on day 35 post initial vaccination. Each serum sample was analysed in two independent neutralisation assays and the mean neutralising antibody titre calculated. Presented are the mean values (Log_{10}), indicated by the line ± S.D., for the

eight animals included in the study. The dotted line illustrates the neutralising capacity threshold in the virus neutralisation assay. *** $p<0.001$ compared to day 0 or indicated time point as determined by one-way ANOVA.

ESM References:

[1] Flodstrom M, Maday A, Balakrishna D, Cleary MM, Yoshimura A, Sarvetnick N (2002) Target cell defense prevents the development of diabetes after viral infection. Nature immunology 3: 373-382

[2] Hankaniemi MM, Laitinen OH, Stone VM, et al. (2017) Optimized production and purification of Coxsackievirus B1 vaccine and its preclinical evaluation in a mouse model. Vaccine 35: 3718-3725

[3] Honkanen H, Oikarinen S, Pakkanen O, et al. (2013) Human enterovirus 71 strains in the background population and in hospital patients in Finland. Journal of clinical virology : the official publication of the Pan American Society for Clinical Virology 56: 348-353
